# Probable Exit Strategy Against COVID-19 of Low Resource Country like Nepal: Open Floor Discussion

**DOI:** 10.31729/jnma.4974

**Published:** 2020-04-30

**Authors:** Bibek Rajbhandari, Minani Gurung, Lisasha Poudel, Archana Shrestha, Biraj Man Karmacharya

**Affiliations:** 1 Nepal Police Hospital, Maharajgunj, Kathmandu, Nepal; 2 Nepal Institute of Development Studies, Kathmandu, Nepal; 3 Kathmandu University School of Medical Sciences, Dhulikhel, Nepal

**Keywords:** *COVID-19*, *lockdown*, *exit strategy*, *Nepal*

## Abstract

Lockdown is essential for containing the spread of SARS-CoV-2. It is the best measure to maintain extreme social distancing which has been effective in controlling the infection and saving lives. But they are causing huge loss economically, disrupting social life and causing distress around the world. Reopening too quickly or too boldly without a goal-oriented strategy could mean a second wave of infection as fierce or even worse as the first. The fundamentals of the virus remain the same - one infected person will, without a lockdown pass it onto three others on average. The consequences of lifting the lockdown are unforeseeable and the stakes are high. Due to the different spectrum of severity with same strain of virus and uncertainty of post lockdown era, lifting the lockdown will be a trial and error approach. Nevertheless, at some point the lockdown has to be lifted. The strategic approach would be innumerable testing, investigations, strong contact tracing, isolation and follow-up. In a low-income country like Nepal, this will mean negotiating a tricky balance between terminating the spread of SARS-CoV-2, and allowing people to recover their livelihoods before they slip into extreme poverty and anguish.

## INTRODUCTION

Coronaviruses are important human and animal pathogens. At the end of 2019, a novel coronavirus was identified as the cause of a cluster of pneumonia cases in Wuhan, a city in the Hubei Province of China. It rapidly spread, resulting in an epidemic throughout China, followed by an increasing number of cases in other countries throughout the world. In February 2020, the World Health Organization designated the disease COVID-19, which stands for coronavirus disease 2019. The virus that causes COVID-19 is designated severe acute respiratory syndrome coronavirus 2 (SARS-CoV-2); previously, it was referred to as 2019-nCoV.^1,2^

## CURRENT SITUATION

Globally, more than three million confirmed cases of COVID-19 have been reported. Since the first reports of cases from Wuhan at the end of 2019, more than 80,000 COVID-19 cases have been reported in China, with the majority of those from Hubei and surrounding provinces. A joint World Health Organization (WHO)- China fact-finding mission estimated that the epidemic in China peaked between late January and early February 2020 and the rate of new cases decreased substantially by early March.^3^

However, cases have been reported in all continents, except for Antarctica, and have been steadily rising around the world.

In Nepal, cases have risen up to 69 confirmed cases out of which 16 have fully recovered and 53 are in isolation in various hospitals. Across the country in 15 different labs 13,414 Real Time Polymerase Chain Reaction (RTPCR) tests have been done. Total of 49,688 Rapid Diagnostic Test (RDT) have been conducted till date and there were 61 positive cases. As of today 21,958 are in quarantine. So far, no deaths have been reported due to COVID-19 in Nepal.^4^

**Figure 1.**
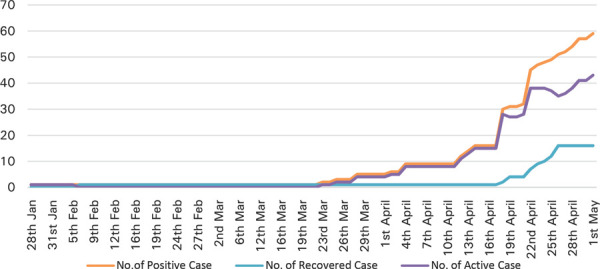
COVID-19; positive recovered and active cases.^4^

**Figure 2.**
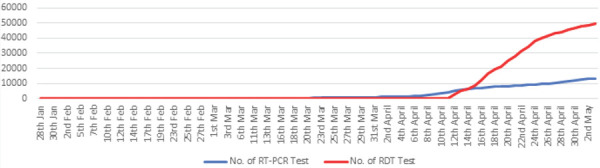
Number of RDT and RT-PCR performed.^4^

**Figure 3.**
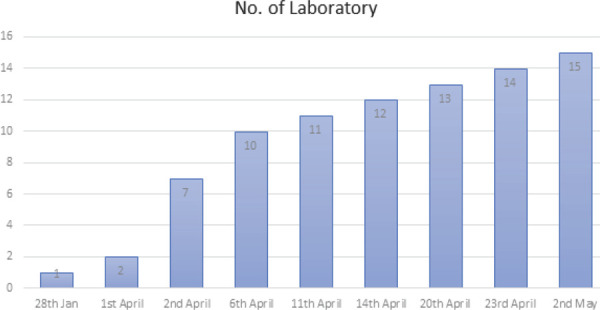
Numbers of Laboratory Involved for RT-PCR Test.^4^

When the first case of COVID-19 was confirmed in Nepal on 25th January 2020, there were 1297 cases in China and zero cases in India, both countries that lies as neighbors to Nepal. On this date, globally COVID-19 has spread to ten countries (China, Japan, Korea, Vietnam, Singapore, Australia, Thailand, United States of America, French Republic) at this point.^5^

Nepal went on a strict lockdown on 24th March 2020, when second confirmed case was reported. At this stage, COVID-19 had already spread to 195 countries across the globe. It was spreading like wildfire and out of control. China's numbers had spiked up to 81,747 and India had gone from zero to 434 cases.^6^

Extreme social distancing was pretty much the only intervention available to help individuals stay healthy, and to break the chain of transmission - giving more vulnerable populations a fighting chance of surviving this pandemic.

But how exactly does a lockdown work? And why is it important for even younger and healthier people, who face a lower risk of severe illness, to remain in their homes as much as possible?

The goal: R<1

The purpose of a lockdown, is to reduce reproduction- in other words, to reduce the number of people each confirmed case infects. The goal is to keep reproduction, or “R,” below one (R<1) - with each case infecting fewer than one other person, on an average.

Epidemiologically speaking the reproduction number (R0) is between 2-3. It signifies herd immunity is achieved, with the immunity of 50-75% of the population. Once effective reproduction number (R) will become lower than (R0) and once R<1, herd immunity is achieved and epidemic declines.^7,8,9^

## ADVANTAGES OF LOCKDOWN

The main motive of lockdown is to confine the virus and its spread. The best outcome of lockdown is the lives saved. Learning from China, who conducted the successful vast extreme lockdown in Wuhan with approximately 11 million people, the cases dropped to zero by 11th March 2020. With many intensive studies and research still undergoing for the vaccine and treatment for COVID-19 the safest measure is social distancing, hand hygiene and respiratory hygiene. Lockdown can benefit at local level, national level and global level. At the local level, fewer people may get infected and hence the health care is not excessively strained. At the national level, it buys critical time required to build the capacity of health care (testing and tracing; protective equipment, case management) to respond the epidemic. At the global level, it will help slow down the pandemic and enabling global resources to be harnessed to deal with the epidemic at the source. Additionally, lockdown is buying more time to be prepared, to find a cure and most importantly keep humans safe.^10^

## DISADVANTAGES

With all its beneficial factors weighing profoundly, nonetheless there is a dark side of the lockdown. It has hugely affected three major fields:

- **Social life:** With social distancing and movement restrictions interfering their regular schedules social life has been disrupted. Basic touch as in handshakes and hugs are now restricted. Communication is now limited to phone calls and video calls. Social gatherings are prohibited. Many religious and family events such as weddings, engagements, weaning ceremonies which are planned a year ahead are cancelled. With lockdown the social life has come to halt with the closing of schools, universities, restaurants and working from home offices. With the lockdown, social life has come to a halt.^11^
- **Economy:** The disease has not remained only a health issue, but also become an economic catastrophe. The economic shock of a disaster propagates downstream to customers through lack of supplies, and upstream to suppliers through lack of demand. The major stock markets are plunging, industries are shutting down, and oil prices are tumbling like never before. Chances are high that the disease will affect more people economically — either causing them to lose their jobs or making them go bankrupt—than through infection.^12^
- **Mental Health:** As the coronavirus pandemic spread rapidly across the world, it is giving birth to a considerable degree of fear, worry and concern in the population at large and among certain groups in particular, such as older adults, health care providers and people with underlying health conditions. The fear of uncertainty creates confusion and anxiety. According to Lancet Psychiatry 2020 the mental health effect might be profound, these are suggestions that suicide rate will rise. Suicide is likely to become a more pressuring concern as the pandemic spreads and has long term effect on general population.^13^
- **Non-COVID health burden:** The lockdown has interfered with the access to the regular health care. With closure of hospitals and health care facilities, and public transportation, population has been devoid of regular health care. Specific populations such as children, pregnant women, and people with chronic diseases will be affected the most. The impact of lockdown will be translated into higher non-COVID morbidity and mortality.

## WHEN IS IT SAFE TO LIFT LOCKDOWN?

While COVID19 spreads fast, it decelerates very slow. That means the control measures must be lifted very slowly and not all at once. Lifting or even loosening lockdown can only be considered when there is a specific and strong strategy. Without any such plans lifting lockdown an explosive outbreak is inevitable.

According to WHO Director General on the briefing of COVID19 there are six criteria for countries as they considering lift restrictions or lockdown:

1. First the transmission must be controlled;
2. Second the health system capacities are in place to detect, test, isolate and treat every case;
3. Third, that outbreak risks are minimalized in special settings like health facilities and nursing homes;
4. Fourth, that preventive measures are in place in workplaces, schools and other places where its essential for people to go;
5. Fifth, that importation risks can be managed;
6. And sixth, that communities are full educated, engaged and empowered to adjust to the “new norm”.

Back to the normal, means the risk of re-introduction and continuation resurgence of the disease. So how can a low resource country like Nepal lift lockdown without cases spiking out of control and beyond our resources.^14^

## THINGS TO CONSIDER

1. **Test, test, test:** Massive testing is the foremost important step in the meticulously planned strategy to lift lockdown. It is the key to detect both infection and immunity. If any one lesson has come out of the rapid spread and sweeping death toll of this coronavirus over the past few months, it's the importance of testing. But how do we know the testing is working? The answer is test positivity rate -- the proportion of tests coming back positive. Dr. Mike Ryan, Executive Director of the WHO's Health Emergencies Programs, said recently that a good benchmark is to have at least 10 negative cases for every one positive case confirmed. That means if a state or country carries out testing and comes back with positive cases of around 9% or under, then its likely that it is testing well. Many countries still don't have sufficient testing capacity-partly because of a shortage of chemical reagents used in tests. International co-operations and other stakeholders should be considered for aid on primers and kits.^15^
  Finally places where the testing is highly positive should be marked as ‘hotspots’ and lockdown should be lifted at the last in these places.
  Testing should be rampant, but prioritized. The testing should be extended and expanded without overwhelming health system. The priority testing should quickly be expanded for high-risk people such as migrant workers returning to Nepal, close household contacts of the returnees, health care workers and close contacts of confirmed COVID-19 cases. Testing for low-risk people should be prioritized in those operating sectors that are essential such as food, medicine and supplies manufacturers and transportation. It is also equally important to learn the sero-prevalence of the community to map-out the relatively ‘safe’ zones. The ‘negative’ test results of ‘RDT’ provides an important information for this.
2. **Strong contact tracing:** Without a vaccine in sight, to safely lift lockdown the government should test, trace and quarantine. Find the hotspot of the virus, find that people who have symptoms actually have virus, and then find people who they've been in contact with and isolate them. Contact tracing should be very efficient. Between manual and tracing apps, apps are more accurate and convenient (for example WHO's tracing app Go.Data) Risk Communication and Community Engagement (RCCE) conceptual model should be applied for an effective contact tracing. If we cannot trace, then it's back to square one.^16^
  Before loosening the lockdown, it is of utmost importance to build contact tracing capacity at the local level and connect the local, provincial and federal level. The use of technologies such as tracing application and cell-phone mobility can be of greatest value to aid identifying the contacts and to monitor the mobility of contacts in real time. For the infectious disease like COVID-19 (with Reproduction rate of 2.5), more than 70% of the contacts has to be traced and contained. The speed and comprehensiveness of contact tracing is equally important to contain the disease.
  - Intensify and know exact surge capacity: This requires proper coordination with multiple levels of government including armed forces. It is important to bring all the scattered data into one common platform such as distribution of foreign returnees, distribution of positive and negative RDT tests, distribution of positive and negative PCR tests, and distribution of health care services. This integrated information will tremendously support to identify local needs and responses including:
  - Local response capabilities: We have to recognize our local resources and manpower in order to be prepared for the surge in infected cases.
  - Control hotspots: Recognizing the true hotspots of infection and apply strict restrictions in mobilization.
  - Country resource allocation: Human resources (front liners) should be allocated and ready for the second wave of the pandemic. ICU setting, number of ventilators and availability of standard PPE should be recorded for calculation of search capacity.
3. **Population demography study:** Population of age above 55 years (geriatric population) who are prone for severity of the disease and have high chances of comorbidity. It was seen in cases of China, Italy, UK and more countries that the severity of the disease and mortality rate was higher in this age group. According to National Population and Housing Census 2011 population of above 55 years old was 11.23% which means around 90% of the population pyramid is below 55 years. To achieve herd immunity, in case of Nepal we can easily achieve it by 60%.^17^
4. **Foreign movement:** According to Department of Immigration of Nepal, total flow of tourists in 2019 were 11,97,191. Among these 35% were from India and China. In the past year during the same months the flow of tourists were 45% via land. According to Department of Foreign Employment, Nepalese going abroad for work are highest among Dhanusha, Mahottari, Jhapa, Siraha, Morang and Saptari. These areas are high in movement and will also see a surge in returnees back to Nepal from various countries. Hence these particular areas must be under careful watch. Testing should be done more here. Lockdown restrictions must be firm and should be lifted only at the last among other areas.^18,19^
5. **Phase wise pattern:** Lifting the lockdown should be in two phases:
  - Initial phase: In the initial phase, we need to analyze and locate the areas with controlled transmission (after testing of high risk populations) and with the health system capacity in place to detect, test, isolate and treat every case. Analyze the areas where there is high density of population above 55 years old. According to the data provided by National Population and Housing Census 2011, the density of geriatric population is high in rural areas compared to urban areas in Nepal. While comparing the three most urban cities of Nepal, (Kathmandu, Bhaktapur and Lalitpur) Kathmandu has 3 times the higher density of geriatric population. Lockdown should be restricted and complete reopening should be prohibited in these areas. The first to reopen will be be the sectors that can ensure better social distancing and other preventive measures such as schools, colleges, recreational centers which involves mobilization of population below 55 years. For the geriatric population in areas where lockdown have been loosened extreme social distancing should still be continued.^20^
  - Late phase: This phase will not be soon until then we buy time. By this time there might be possible chances of a vaccine. The first ones to get the vaccine should be the immunocompromised, front liners (medical staffs, police officers) and the geriatric population. As we enter this phase there is high probability of developed herd immunity in the community.

*In between the above two phases, monitoring of cases increasing and resources available should be done. Cases should be below surge capacity level. If seen to be rising, or reaching neck to neck level lockdown has to be resumed immediately.^21,22,23^

## COUNTRY WISE SITUATION:

**New Zealand:** On 23rd March 2020 New Zealand entered one of the world's strictest lockdown for five weeks. When it comes to what worked, New Zealand had some advantages in tackling the virus. It had the benefit of time -- New Zealand confirmed its first case of coronavirus on February 28, 2020.But the real key to New Zealand's success appears to be an approach that could be applied anywhere - testing widely and good contact tracing.

**Italy:** Italy, with the most COVID-19-linked deaths in Europe, is keeping the lockdown intact although it lifted restrictions on two categories of shops - stationers and children's clothes - on April 14, 2020.

**France:** After being hit by the outbreak France had one of the highest number of cases. After being in lockdown for 4 weeks with allowing the citizens to move outside only once. Now the Prime Minister has extended the lockdown up to 8 weeks and planning of reopening slowly. The coming weeks will be used to obtain better and more equipment, masks and tests.

**Britain:** The lockdown that started from 23rd March, 2020 is still in ongoing process. The government believes relaxing the restrictions would help in increasing the outbreak. Lack of masks, gloves and a major shortfall in testing Britain has extended the lockdown until further notice.

**Germany:** As being applauded for having the outbreak under control Germany eased the restrictions with masks mandatory in shops and on public transport.

**China:** Earlier this month China lifted the lockdown of Wuhan after 76 days. On April 15, the city's vice mayor said it aimed to fully restore rail, flight and freight operations by the end of April. Prevention measures remain elsewhere, including Beijing.

**India:** Lockdown is extended the until May 3 in India, after closing the country for a month. The government will allow some industries, such as farming and construction in rural areas, to open after April 20,2020.

**Pakistan:** Extended the lockdown on April 14 by two weeks, said some industries would reopen in phases, starting with construction and export industries, such as garments. It is switching to a so-called ‘smart- lockdown’ in which it will instrument targeted tracking and tracing of cases supported by Pakistan's Inter-Services Intelligence service.

**Japan:** A nationwide lockdown was declared as the country's scenario worsened with the outbreak. Despite the first case, more than three months ago, Japan is conducting tests at a much slower pace than other countries gaining criticism nationwide.

The above countries have or are in process of reopening lockdown. The planning, organizing and challenges faced by these various countries can be taken as lessons.^24,25,26,27,28,29,30,31,32^

## Conflict of Interest

**None.**
